# Norepinephrine and Serotonin Can Modulate the Behavior of the Probiotic *Enterococcus faecium* NCIMB10415 towards the Host: Is a Putative Surface Sensor Involved?

**DOI:** 10.3390/microorganisms10030487

**Published:** 2022-02-22

**Authors:** Rossella Scardaci, Francesca Bietto, Pierre-Jean Racine, Amine M. Boukerb, Olivier Lesouhaitier, Marc G. J. Feuilloley, Sara Scutera, Tiziana Musso, Nathalie Connil, Enrica Pessione

**Affiliations:** 1 Laboratory of Microbial Biochemistry and Proteomics, Department of Life Sciences and Systems Biology, University of Turin, Via Accademia Albertina 13, 10123 Torino, Italy; francescabietto@gmail.com (F.B.); enrica.pessione@unito.it (E.P.); 2 Laboratory of Microbiology—Bacterial Communication and Anti-infectious Strategies, University of Rouen Normandy, 27000 Evreux, France; racinepierrejean@gmail.com (P.-J.R.); amine.boukerb@univ-rouen.fr (A.M.B.); olivier.lesouhaitier@univ-rouen.fr (O.L.); marc.feuilloley@univ-rouen.fr (M.G.J.F.); nathalie.connil@univ-rouen.fr (N.C.); 3 Laboratory of Immunology, Department of Public Health and Pediatric Sciences, University of Turin, Via Santena 9, 10126 Torino, Italy; sara.scutera@unito.it (S.S.); tiziana.musso@unito.it (T.M.)

**Keywords:** biofilm, adhesion, TER, dendritic cells, TNF-α, IL-10, hormone sensor

## Abstract

The human gut microbiota has co-evolved with humans by exchanging bidirectional signals. This study aims at deepening the knowledge of this crucial relationship by analyzing phenotypic and interactive responses of the probiotic *Enterococcus faecium* NCIMB10415 (*E. faecium* SF68) to the top-down signals norepinephrine (NE) and serotonin (5HT), two neuroactive molecules abundant in the gut. We treated *E. faecium* NCIMB10415 with 100 µM NE and 50 µM 5HT and tested its ability to form static biofilm (Confocal Laser Scanning Microscopy), adhere to the Caco-2/TC7 monolayer, affect the epithelial barrier function (Transepithelial Electrical Resistance) and human dendritic cells (DC) maturation, differentiation, and cytokines production. Finally, we evaluated the presence of a putative hormone sensor through in silico (whole genome sequence and protein modelling) and in vitro (Micro-Scale Thermophoresis) analyses. The hormone treatments increase biofilm formation and adhesion on Caco-2/TC7, as well as the epithelial barrier function. No differences concerning DC differentiation and maturation between stimulated and control bacteria were detected, while an enhanced TNF-α production was observed in NE-treated bacteria. Investigations on the sensor support the hypothesis that a two-component system on the bacterial surface can sense 5HT and NE. Overall, the data demonstrate that *E. faecium* NCIMB10415 can sense both NE and 5HT and respond accordingly.

## 1. Introduction

The genus *Enterococcus* has been the object of several reports questioning its safety when used as a probiotic during the last two decades [1,2,3]. In addition to starter strains employed in food production [4], biocontrol strains [5], putative [6,7,8] and approved probiotics commercially available for human and animals’ treatment [2,9], several hospital strains displaying both antibiotic resistance and production of virulence factors have been isolated so far [10,11]. These are among the most diffused opportunistic nosocomial pathogens involved in severe diseases such as urinary infections, endocarditis, bacteremia, and central nervous system infections [12]. In particular, these bacteria bear in their genomes multidrug resistance genes [13,14] and the so-called pathogenicity island, which encodes for several virulence factors, such as proteases, tissue-targeted toxins, or adhesive molecules that mediate aggregation and adhesion to the host [11]. These factors, when present together, constitute a significant threat to human health. Furthermore, both can be transmitted by horizontal gene transfer (HGT) [15]. Hence, a “safe” strain could acquire them from a pathogen, thus turning into a “dangerous” one. In controversial bacteria, as enterococci are, this aspect is particularly challenging, especially in the gut habitat where they usually live [14,16].

A further concern is that environmental conditions can also affect the expression of genome-encoded virulence factors, as demonstrated in *Enterococcus faecalis* [10,17,18]. Since probiotic features can also be affected by environmental modulation of gene expression, investigation on how enterococcal performances are affected by the environment they typically inhabit is a critical concern. In this regard, the human gut becomes an intriguing ecological niche that provides a broad spectrum of stressors. For example, host-derived signals in the gut, such as norepinephrine (NE) and serotonin (5HT), have been reported to trigger the virulence of some intestinal pathogens including *E. faecalis* [19,20,21], but those conditions can also modify the probiotic properties of exogenously administered enterococci [21]. Previous research tested how NE and 5HT can influence the basal physiological features (growth and protein profiles, resistance to bile salts, and antibiotics) of the probiotic *E. faecium* NCIMB10415 (*E. faecium* SF68) and its capability to auto-aggregate and form biofilm [22,23]. Curiously, the proteomic results of 5HT treatment suggested that the effects triggered by this molecule could be mediated by a sensor, primarily described as human signal receptors in prokaryotes [24]. Indeed, bacteria have evolved different response mechanisms to host-derived signals, including Two Component Systems (TCS), firstly described for catecholamines in *E. coli* [25,26]. Considering these overall interesting findings, the present study aimed to evaluate the effects of the hormone treatments on the bacterial–host relationship. This interaction mainly involves communication between bacteria and epithelial cells of the intestinal mucosa, but also cross talk with the gut immune system (GALT), especially mediated by dendritic cells (DC) whose dendrites protrude into the intestinal lumen, thus coming into direct contact with gut microbiota [27,28]. (Considering that GALT represents a consistent part of our overall immune system, depicting possible effects of the (treated and untreated) strain in study on its performances is of primary importance to corroborate the growing evidence that a healthy immune status is dependent upon a healthy gut microbiota. In particular, Enterococci, both *E. faecalis* and *E. faecium*, have been shown to play a crucial role in immune modulation at the gut level [29,30]. Thus, we determined if the treatment with both hormones affected *E. faecium* NCIMB10415 ability to colonize the gut mucosa (biofilm formation and adhesion on Caco-2/TC7 cells) or could affect the intestinal barrier (by measuring the transepithelial electrical resistance—TER—as an index of permeabilization of the epithelial cell membrane). Additionally, we attempted to evaluate if the treatments impacted the immune system (IS) responses to *E. faecium* NCIMB10415 by challenging human DCs with our strain. Finally, in silico analyses and Micro-Scale Thermophoresis have been used to evaluate the implication of a putative sensor for the detected modifications.

## 2. Materials and Methods

### 2.1. Media and Growth Conditions

*E. faecium* NCIMB10415 (*E. faecium* SF68) was grown at 37 °C in static conditions in a rich Chemically Defined Medium (CDM) to ensure highly reproducible results, as reported elsewhere [22]. 100× solutions of NE (L-Norepinephrine (+)-bitartrate salt monohydrate—Sigma Aldrich, St. Louis, MO, USA) and 5HT (serotonin hydrochloride—Sigma Aldrich) were freshly prepared in CDM, filter sterilized and diluted to obtain a final concentration that would reflect over secretion in the gut, 100 µM and 50 µM, respectively (for further details see [22,23]). For each experiment, except for biofilm formation, treatments, and control conditions, bacteria were harvested at late exponential phase (~4 h, see [22,23]).

### 2.2. Cell Lines

Human enterocyte-like Caco-2/TC7 (colon adenocarcinoma cells) were grown in 4.5 g/L glucose Dulbecco’s Modified Eagle Medium (DMEM, Lonza, Basel, Switzerland) supplemented with 15% heat-inactivated fetal bovine serum (FBS, Sigma Aldrich), 1% penicillin/streptomycin (Sigma Aldrich), and 2 mM glutamine (Sigma Aldrich). Cells were maintained in a humidified incubator at 37 °C in 5% CO_2_ and 95% air atmosphere, and the medium was replaced thrice a week. Cells were passed after reaching approximately 90% of confluence. Monocytes were isolated from peripheral blood mononuclear cells (PMBC) obtained from healthy donor buffy coats (through the courtesy of the S.C. Centro Produzione e Validazione Emocomponenti, Torino, Italy) by Ficoll (Biochrom) gradient (Pharmacia Fine Chemicals, Uppsala, Sweden). CD14^+^ were isolated from PBMCs by immune-magnetic selection with CD14 microbeads and magnetic separation columns (MACS monocyte isolation kit from Miltenyi Biotec, Bergisch Gladbach, Germany). This procedure yielded at least 98% pure monocyte population, as assessed by fluorescence-activated cell sorter analysis (FACSCalibur, BD Biosciences, Franklin Lakes, NJ, USA). To obtain monocyte-derived DCs, monocytes were cultured for 5 days at 10^6^ cells/mL in RPMI 1640 medium (Sigma Aldrich) supplemented with 10% FBS (Sigma Aldrich), 50 ng/mL GM-CSF (Sigma Aldrich) and 20 ng/mL IL-4 (Sigma Aldrich) [31].

### 2.3. Confocal Laser Scanning Microscopy (CLSM) Biofilm Formation

Overnight precultures were diluted to OD_600_ 0.1 (10^8^ CFU/mL) with or without the molecules, and 500 μL of each treatment were seeded in triplicate on a glass flat-bottom 24-wells plate (Sensoplate, Greiner Bio-one, Stonehouse, UK). Bacteria were grown for 24 and 48 h at 37 °C (for the measurement at 48 h, the medium and the molecules were replaced after 24 h without perturbing the bacterial biomasses in the biofilms), after which planktonic cells were removed by three washings with sterile NaCl 0.9%. Next, the biofilms were stained with 5 μM of SYTO^®^ 9 green-fluorescent nucleic acid stain (ThermoFisher Scientific, Waltham, MA, USA) for 15 min in the dark, then washed thrice and observed by CLSM (LSM710, Zeiss, Oberkochen, Germany) with at least 4 captures for each well [21]. The results are a medium of ten biological replicates.

### 2.4. Adhesion

Caco-2/TC7 cells were seeded in 24-well culture plates treated for tissue culture and used at the confluence (10^6^/well). On the experiment day, *E. faecium* NCIMB10415 was harvested by centrifugation (10,000× *g*, 10′, RT) at the late exponential phase after incubation with the molecules and in control conditions. The pellets were concentrated to 10^10^ CFU/mL in DMEM without FBS and antibiotics, diluted 1:100, counted on plates, and applied on confluent cells monolayers—previously washed with saline solution to remove the antibiotics—for 2 h (MOI 1:100 eukaryotic cells: bacteria). Caco-2/TC7 were washed twice with NaCl 0.9% to remove non-adherent bacteria, and 500 μL of 0.1% Triton 100-X was added to disrupt the cells. Then, lysates were serially diluted and plated on BHI agar to count the number of adherent bacteria.

### 2.5. Transepithelial Electrical Resistance (TER)

The effect of NE and 5HT on the modulation of the TER induced by *E. faecium* NCIMB10415 was assessed as described before [8]. In brief, cells were grown on inserts (3 μm pore size) for 21 days to ensure epithelial differentiation. On the experiment day, treated and untreated bacteria recovered after 4 h of growth were concentrated to 10^10^ CFU/mL in DMEM without FBS and antibiotics and diluted to 10^8^ CFU/mL. Microbial cells were deposited on Caco-2/TC7 previously washed twice with saline solution to remove traces of antibiotics and incubated till 20 h. TER was measured using a Millicell Electrical Resistance system (Millipore, Bedford, MA, USA) at T_0_, T_4_, T_16_ and T_20_, and results were reported as % of initial TER.

### 2.6. DCs Stimulation

To study the effects of the treated *E. faecium* NCIMB10415 on immune cells, we stimulated DCs for 40 h with HK bacteria and Cell-Free Supernatants (CFSs). Late exponential phase treated and untreated bacterial cultures were centrifuged at 10,000× *g* for 10 min and the CFSs were collected. To prepare the heat-killed (HK) sample, bacterial cells were washed twice, resuspended in PBS to 10^9^ CFU/mL and heated for 30 min at 90 °C. Complete loss of cell viability was verified by monitoring colony formation on agar plates. On the day of the experiment, HK bacteria were diluted in RPMI 1640 medium to obtain a bacteria/host ratio 10:1 and deposited on immature DCs, previously seeded at the density of 10^6^ cells/mL. DCs were also challenged with LPS (100 ng/mL) as costimulatory molecule and with a combination of LPS and HK bacteria (100 ng/mL LPS and HK bacteria MOI 10:1). CFSs obtained by centrifugation were neutralized with 1M-NaOH to pH 7 and sterilized by filtering at 0.22 μm and stored at −20 °C until use. For the experiment, CFSs were added to the DC culture medium at a concentration of 10–30% *v*/*v* and incubated with the immature DCs (10^6^ cells/mL). After 40 h incubation, cells and cell supernatants were collected for flow cytometric and cytokine analysis, respectively. Immature DCs incubated with RPMI 1640 medium were used as negative control.

### 2.7. Flow Cytometric Analysis and Cytokine Quantification in Culture Supernatants

DCs were collected after the indicated time of infection and preincubated for 30 min at 4 °C in PBS containing 2% goat serum plus 0.2% sodium azide and washed twice with 1% bovine serum albumin (BSA). Then, cells were incubated for 30 min at 4 °C with anti-human CD14-FITC (clone REA599 1:50), anti CD1a-APC (clone REA736 1:50), anti CD80-PE (clone REA661 1:50), anti-CD-83-VioFITC (clone REA714 1:100), and the specific REA control (S) antibodies human IgG1 (all purchased from Miltenyi Biotec, Bergisch Gladbach, Germany). Flow cytometry analysis was performed using FACSCalibur (BD Biosciences, NJ, USA) and FlowLogic software (Miltenyi Biotec).

The culture supernatants of DC were assayed to evaluate the pro-inflammatory cytokines TNF-α (DY210, assay range: 15.6–1000 pg/mL), IL-6 (DY206, assay range: 9.4–600 pg/mL), IL-8 (CXCL8) (DY208, assay range: 31.2–2000 pg/mL), and the anti-inflammatory cytokine IL-10 (DY217B, assay range: 31.2–2000 pg/mL) by DuoSet ELISA kits purchased from R&D Systems (R&D System, Minneapolis, MN, USA) and used according to the manufacturer’s instructions.

### 2.8. Genome Sequencing and Annotation

*E. faecium* NCIMB10415 was cultured in CDM medium O/N at 37 °C. Genomic DNA was extracted using the GeneJet genomic DNA purification kit as recommended by the manufacturer (ThermoFisher Scientific), then quality and quantity were assessed using the double-stranded DNA (dsDNA) high-sensitivity kit on a Qubit fluorometer (ThermoFisher Scientific) and 1% agarose gel electrophoresis. Sequencing libraries were prepared using the Nextera XT reagent kit (Illumina, San Diego, CA, USA) according to the manufacturer’s recommendations. Sequencing was performed by the genomics platform of the Laboratory of Microbiology—Bacterial Communication and Anti-infectious Strategies (CBSA) (Rouen Normandy University, Evreux, France) using an Illumina MiSeq system with a 2 × 250-bp paired-end read protocol. Default parameters were used for all software tools unless otherwise noted. TrimGalore v.0.6.2 [32] was used for reads trimming, and read quality was checked with FastQC v.0.11.9 [33]. Raw paired-end reads were assembled de novo with Unicycler v.0.4.7 [34], and assembly metrics were calculated using QUAST v.5.0.0 [35]. Structural gene prediction and functional annotation were carried out using the Prokka pipeline v.1.14.0 [36].

### 2.9. Putative Sensor Sequence Screening and Alignment

We used the Prokka annotation (faa file) to retrieve the putative adrenergic receptor in *E. faecium* NCIMB10415 genome sequence, based on sequence identity/coverage comparison to QseC from *E. coli* K12 and VicK (WalK) previously found in *E. faecalis* [21]. CLUSTALX2 v.2.1 was used for multiple sequence alignments of the obtained protein sequence and that from *E. coli* K12, *E. faecalis* OB15, *E*. *faecalis* ATCC19433^T^ and *E. faecium* NCTC7171^T^. The alignment file was visualized with Jalview 2 [37].

### 2.10. Structure Modeling and Docking

The sequence annotated in the *E. faecium* NCIMB10415 genome as VicK (WalK) was analyzed with the online server PSIPRED to predict its secondary structure and subcellular localization (http://bioinf.cs.ucl.ac.uk/psipred (accessed on 20 October 2020)) [38]. Comparative 3D modeling was realized with Raptor X (http://raptorx.uchicago.edu/StructurePrediction/ (accessed on 17 November 2020)), GalaxyWEB (http://galaxy.Seoklab.Org/ (accessed on 17 November 2020)) [39] and MODELLER (https://salilab.org/modeller/ (accessed on 17 November 2020)) [40]. The models generated by MODELLER were submitted to the energy function DOPE, and QMEAN (https://swissmodel.expasy.org/qmean/ (accessed on 24 November 2020)) was used to validate a model among the others [41].

Potential ligand interactions (NE, 5HT, phentolamine, and propranolol) were investigated in silico by the molecular docking technique using the predicted structure of the putative adrenergic sensor of *E. faecium* NCIMB10415. Essential hydrogen atoms, Gasteiger charges, and solvation parameters were added with the aid of AutoDock tools. Affinity (grid) maps of 80- by 80- by 80-Å grid points and 0.375-Å spacing were generated using the Autogrid program [42]. AutoDock parameter set- and distance-dependent dielectric functions were used to calculate the van der Waals and electrostatic terms, respectively. Docking simulations were made using the Genetic Algorithm (GA). The structure of NE (CID: 439260), 5HT (CID: 160436), phentolamine (CDI: 5775), and propranolol (CID: 4946) were downloaded from PubChem (http://www.ncbi.nlm.nih.gov/pcc (accessed on 30 November 2020)) in SDF and converted to PDB through PyMOL (http://www.pymol.org/ (accessed on 30 November 2020)).

### 2.11. VicK (WalK) Expression and Purification

To express *E. faecium* NCIMB10415 VicK (WalK) in *E. coli*, a synthetic codon-optimized gene was prepared and cloned into the NdeI and BamHI restriction sites of the pET-15b vector, which carries an N-terminal His•Tag^®^ and resistance to Amp (GenScript Biotech, Piscataway, NJ, USA). Competent *E. coli* BL21 were then transformed with this vector and induced with 0.5 mM IPTG for 4 h in agitation (100 rpm) and 25 °C. The cells were cooled in ice, then harvested by centrifugation at 4000× *g* for 20 min at 4 °C and washed with 20 mM Tris-HCl pH 8.0. Next, cells were lysed by sonication and clarified by centrifugation at 4000× *g* for 20 min. Finally, the protein was extracted with 1% dodecyl maltoside (DDM, Applichem Reagents, Darmstadt, Germany) and purified by Immobilized Metal Affinity Chromatography (IMAC). In detail, a 1 mL HiTrap TALON^®^ crude column (Cytiva, Marlborough, MA, USA) was preconditioned with seven volumes of BW buffer (50 mM sodium phosphate buffer pH 7, 300 mM NaCl, 5% glycerol, 20 mM imidazole pH 8, 1% DDM), then the whole lysate was loaded at 0.1 mL/min and washed with 20 volumes of BW buffer at 0.5 mL/min. To increase its purity, VicK (WalK) was step-eluted with an increasing concentration of imidazole (Sigma-Aldrich) as follows: BW Buffer was supplemented with 60/80/100/120/150 mM of imidazole, each solution was applied for 2 column volumes at 0.25 mL/min, and 0.5 mL fractions were collected (total of 20). The protein was concentrated using a Vivaspin 20 centrifugal concentrator (Sartorius, Göttingen, Germany; 50-kDa molecular mass cutoff) at 4 °C and stored at −80 °C.

### 2.12. Micro-Scale Thermophoresis (MST)

Micro-Scale Thermophoresis was performed as described previously [43], with few modifications. VicK (WalK) was labeled using the RED-NHS labeling kit (NanoTemper Technologies, München, Germany). The labeling reaction was performed according to the manufacturer’s instructions in the supplied labeling buffer, applying a concentration of 15 µM protein (molar dye: protein ratio ~6:1) at room temperature for 30 min. The excess of dye was removed by washing (50 mM Tris-HCl pH 7, 300 mM NaCl, 5% glycerol, 0.5% Tween-20) in Amicon^®^ Ultra Centrifugal Filters (Millipore) at 14,000× *g* for 20 min, for three times. The dye/protein ratio was determined using photometry at 650 and 280 nm. VicK (WalK) was adjusted to 7.5 nM with the same buffer. A series of 16 NE and 5HT 1:1 dilutions was prepared in the identical buffer, producing ligand concentrations ranging from 5 mM to 152.6 nM. For thermophoresis analysis, each ligand dilution was mixed with one labelled sensor volume, leading to a final concentration of VicK (WalK) of 3.75 nM and final ligand concentrations at half of the ranges above. Approximately 4 µL of each solution was filled into Monolith NT standard treated capillaries (NanoTemper Technologies GmbH). Thermophoresis was measured using a Monolith NT.115 pico instrument (NanoTemper Technologies GmbH) at room temperature with 5-s/30-s/5-s laser off/on/off times, respectively. Instrument parameters were adjusted with 50% LED power and 60% MST power. Data from three independently pipetted measurements were analyzed (NT.Analysis software version 1.5.41; NanoTemper Technologies). Following data analysis, the thermophoresis signals were fitted to the formula for K_D_ from the law of mass action:

$$f(c)=\frac{\mathrm{unbound}+(\mathrm{bound}\;-\;\mathrm{unbound})\;\times \;(\left[\mathrm{VicK}\right]{+c+K}_{D}\;-\;\sqrt{((\left[\mathrm{VicK}\right]{+c+K}_{D}{)}^{2}\;-\;\left(4\;\times \;\left[\mathrm{VicK}\right]\;\times \;c\right)})}{2\;\times \;[\mathrm{VicK}]\;}$$

where f(c) is the observed thermophoresis signal, “unbound” is the signal in the absence of ligand (normalized to 0), “bound” is the signal at infinite ligand concentration, c is the concentration of ligand in the same units as VicK (WalK) concentration, and K_D_ is the dissociation constant.

### 2.13. Statistical Analysis

All experiments were performed at least three times independently. Statistical data analyses were carried out with GraphPad Prism 9 using a two-tailed *t*-test. Significance was considered at **** *p* ˂ 0.0001, *** *p* ˂ 0.001, ** *p* ˂ 0.01, * *p* ˂ 0.05. Data are expressed either as mean ± standard error (SEM) or ± standard deviation (SD).

## 3. Results

### 3.1. Biofilm

**Figure 1:**
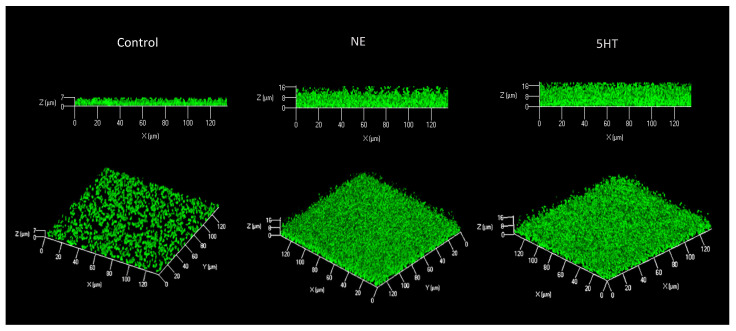
Effect of the treatment of *E. faecium* NCIMB10415 with 100 µM NE and 50 µM 5HT for 48 h on biofilm formation on glass surface, measured by CLSM. Both treatments increase the biofilm volume and thickness.

Our group already assessed the biofilm formation ability of *E. faecium* NCIMB10415 with the crystal violet method in control and stimulated conditions, and both molecules caused a significant enhancement of biomasses after the treatment [22,23]. The CLSM technique was chosen to validate the results obtained with the CV staining since this method is not considered precise. Biofilms were grown for 24 h (results not shown as no significant differences were observed) and 48 h (medium and molecule replacement) on glass surfaces, then their biovolumes, average and maximum thickness were analyzed. Figure 1 and Table 1 illustrate and summarize how NE and 5HT affected our strain’s capability to form biofilms. The hormones have augmented all the three parameters; in detail, the treatment with 100 µM NE enhances the three of them by about 50%, while 50 µM 5HT increases twofold the biovolume and the maximum thickness with respect to the control, while the average thickness is augmented by about 40%. These results strongly confirm the CV assay outcomes concerning the boosting effect of the molecules and its measurement; indeed, 5HT shows a more intense effect compared to NE, precisely as demonstrated with the CV test [22,23].

### 3.2. Adhesion on Caco-2/TC7 Cells

**Figure 2:**
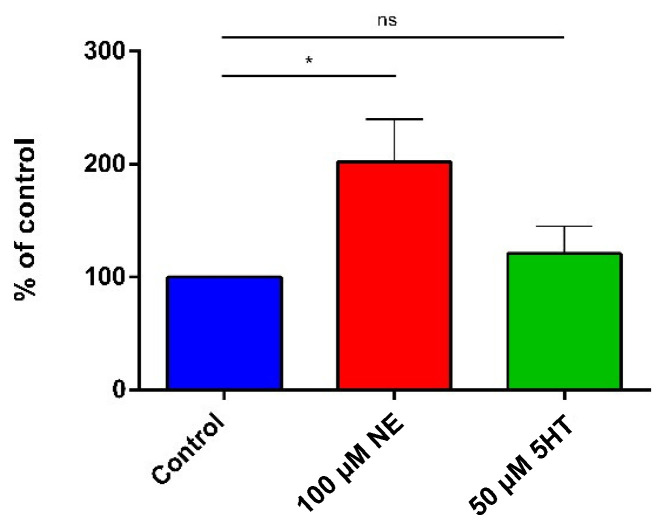
*E. faecium* NCIMB10415 adhesion on Caco-2/TC7 cells in control condition (blue) or stimulated with 100 μM NE (red) or 50 μM 5HT (green). Treatment with 100 μM NE increases the bacterial adhesion. The results are represented as % of control ± SEM. * *p* < 0.05; ns: not significant.

As it is a predictive sign of the probiotic behavior, the adhesion on Caco-2/TC7 cells (a reliable in vitro model of adhesion on mucosal gut tissues [44,45,46]) was investigated by applying hormone-treated and untreated *E. faecium* NCIMB10415 on monolayers for 2 h and then counting adherent bacteria. As illustrated in Figure 2, NE doubled up the capacity to adhere to this model of enterocytes, while 5HT enhanced the adhesion by about 30%; however, this modulation was not significant.

### 3.3. Effect on the Epithelial Barrier Function (TER on Differentiated Caco-2/TC7 Cells)

**Figure 3:**
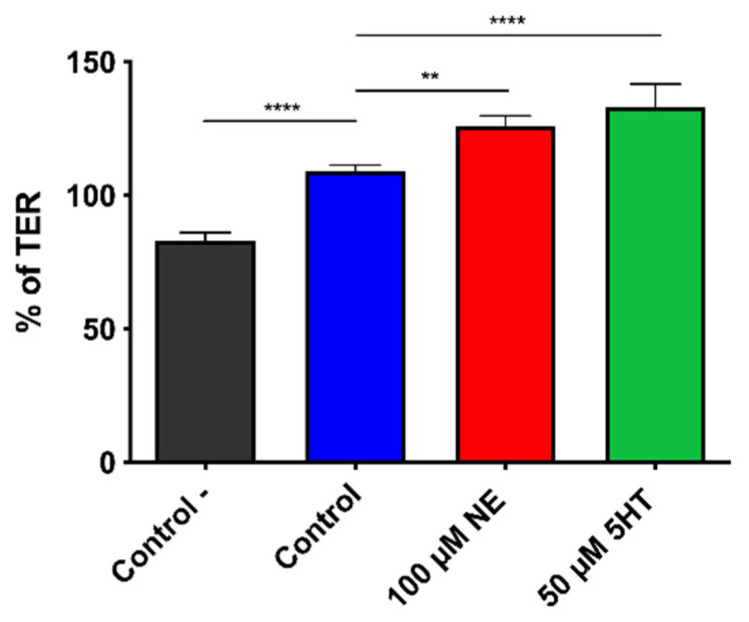
Modulation of Caco-2/TC7 TER after 20 h exposure to *E. faecium* NCIMB10415 in control conditions (blue) or treated with 100 μM NE (red) or 50 μM 5HT (green). Both treatments of the bacteria improve the TER compared to the control condition. In the bar chart is presented also the % of initial TER for the untreated Caco-2/TC7 cells as ‘Control–’ (black). Results are expressed as % of initial TER ± SEM. **** *p* ≤ 0.0001, ** *p* < 0.01.

NE and 5HT modulation of the probiotic efficacy in optimizing the epithelial barrier function was studied, after 4, 16 and 20 h of treatment on differentiated Caco-2/TC7, with the measurement of the TER. Even if the probiotic started slightly to enhance the TER values at T_4_, the differences observed were not significant till 20 h of treatment (Figure 3 and Supplementary Figure S1). Moreover, the treatment with both molecules boosted its capability to modulate the integrity of the intestinal barrier. Specifically, at T_20_ the TER was increased by about 30%, 50%, and 60%, respectively, by the untreated, 100 μM NE and 50 μM 5HT treated bacteria compared to the negative control.

### 3.4. Immune Modulation

To assess the possible immune modulation exerted by 5HT- and NE-treated bacterial cells on DC, we tested the effects of CFSs and living bacteria after growth in stimulated and control conditions. *E. faecium* NCIMB10415 CFSs did not significantly affect DC maturation (d., and the experiment performed with living bacterial cells was unsuccessful since acidification produced during lactic fermentation damaged the DC. Therefore, we decided to stimulate dendritic cells with hormone-treated and untreated HK bacteria. After 40 h of coincubation, DCs and their supernatants were collected for flow cytometric and cytokine analysis, respectively.

#### 3.4.1. DC Differentiation and Maturation

**Figure 4:**
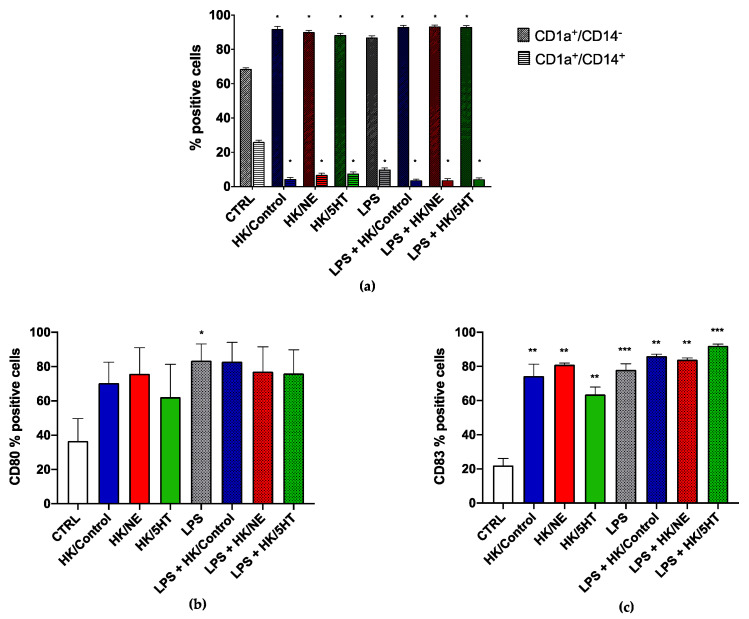
DC differentiation and activation after 40 h incubation with HK/Control (blue), HK/NE (red) and HK/5HT (green) (**a**) % of positive cells ± SEM displaying CD1a^+^/CD14^−^ (oblique-line pattern) and CD1a^+^/CD14^+^ (straight-line pattern) phenotype as markers of differentiation; (**b**) % of CD80 and (**c**) % of C83 positive cells as markers of maturation (LPS cotreatment is presented in dot pattern). Untreated DCs represent the negative control (CTRL) *** *p* ≤ 0.001, ** *p* < 0.01, * *p* ≤ 0.05 to CTRL.

We analyzed the expression of DC differentiation and maturation markers after challenging DC with HK/Control, HK/NE or HK/5HT, alone and in the presence of the costimulatory molecules (LPS). The differentiation of DC was assessed by flow cytometry using the DC marker CD1a and the monocyte marker CD14, whereas the markers CD80 and CD83 were evaluated to ascertain DC maturation. The overall results are summarized in Figure 4. As expected, in the control DC, we found a high percentage of CD1a^+^/CD14^−^, (about 70%), a low percentage of CD1a^+^/CD14^+^ (25%), and the absence of CD1a^−^/CD14^+^ (less than 2%). In the presence of all the tested stimuli, we observed a significant increase in the percentage of CD1a^+^/CD14^−^, in parallel with a decrease in CD1a^+^/CD14^+^, compared to control conditions (Figure 4a). We next analyzed both CD80 and CD83 expression in terms of the percentage of positive cells as markers of maturation of DC. As shown in Figure 4b, a similar increase in the percentage of CD80-positive cells over control DC was observed in both HK/Control, HK/NE, and HK/5HT-treated DCs. CD80 expression was increased to the same extent following LPS stimulation and was not affected by costimulation with bacteria. Similar results were obtained for the percentage of CD83-positive cells (Figure 4c).

#### 3.4.2. Cytokine Quantification in Culture Supernatants

**Figure 5:**
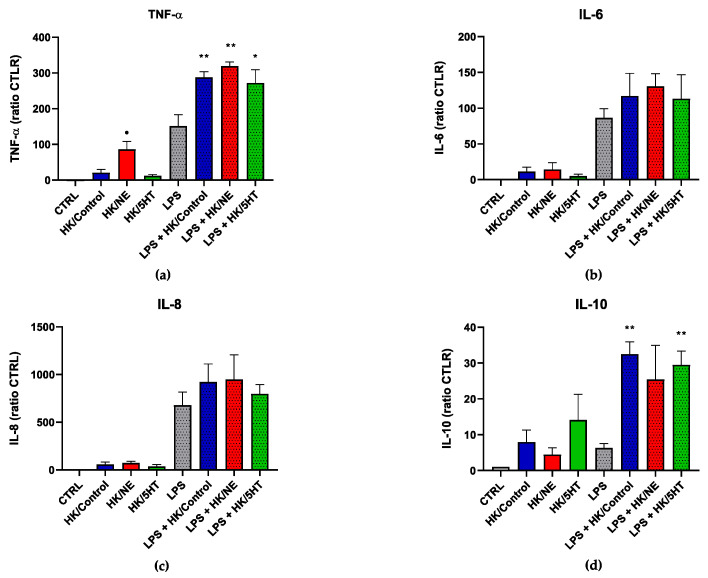
Cytokines’ quantification in DCs culture supernatant after 40 h incubation with HK/Control (blue), HK/NE (red) and HK/5HT (green). Untreated DC supernatants represent the negative control (CTRL). Results are expressed as ratio of CTRL ± SEM. (**a**) TNF-a; (**b**) IL-6; (**c**) IL-8; (**d**) IL-10. • *p* ≤ 0.05 to HK/Control; ** *p* < 0.01, * *p* ≤ 0.05 to LPS.

The effect of control, 100 µm NE and 50 µM 5HT-treated HK bacteria on DC cytokine output was then investigated. Due to high variability among samples (originated from different donors), the results are expressed as a ratio of control (CTRL) conditions. Regarding pro-inflammatory cytokines, Figure 5 shows that the challenge with HK/Control, HK/NE and HK/5HT alone induced a slight production of TNF-α, IL-6, and IL-8 compared with unstimulated DC. However, the amounts of all three cytokines are lower than those obtained in LPS-treated DC, which are 15.1 ± 3.2 ng/mL, 10.5 ± 2.3 ng/mL, and 70.6 ± 14.2 ng/mL, respectively, for TNF-α, IL-6, and IL-8. The simultaneous exposure to HK/Control and LPS did not significantly impact LPS-induced IL-6 and IL-8, while an increase in TNF-α was observed and TNF-α production is enhanced by about two-fold by bacteria treated with NE (Figure 5a). 5HT treatment appears to have no significant effects on cytokine production.

As far as the anti-inflammatory cytokine is concerned, HK/Control, HK/NE, or HK/5HT booster levels of IL-10 are in a way similar to LPS stimulation. HK/NE seems to decrease, and HK/5HT apparently enhances IL-10 production compared to HK/Control stimulation, although the difference is not significant. An additive-like pattern of IL-10 production was observed with the simultaneous stimulation of LPS with HK/Control, HK/NE, or HK/5HT (Figure 5d).

### 3.5. In Silico Analyses of a Putative Sensor

Using the whole genomic sequence data, we screened *E. faecium* NCIMB10415 for a sensor homologous to QseC, the *E. coli* adrenergic sensor, and VicK (Walk) from *E. faecalis* [21]. Based on Prokka annotation, we identified a protein sequence annotated as “Sensor histidine kinase WalK”. BLASTP with QseC from *E. coli* K12 strain indicated 29% identity and 49% similarity values.

**Figure 6:**
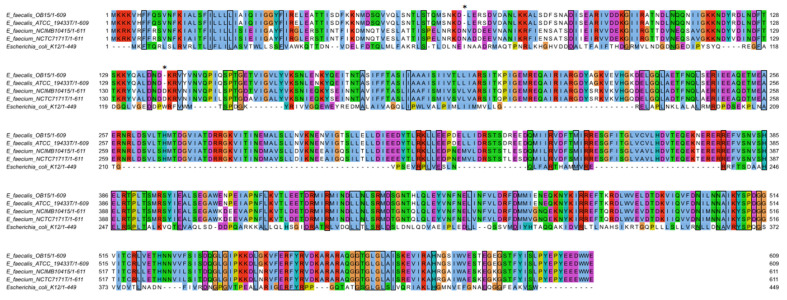
Multiple sequence alignment of *E. faecium* NCIMB10415’s VicK (WalK) amino acid sequence with QseC from *E. coli* strain K-12 and similar sequences from *E. faecalis* and *E. faecium*. Shared amino acids are boxed. Asterisks indicate indels at positions -70N and -140D by comparison to the *E. faecalis* sequences.

Multiple sequence alignment confirmed that VicK (WalK) from *E. faecium* NCIMB10415 was conserved compared to similar sequences from *E. faecalis* strains (Figure 6). Interestingly, two indels were observed at positions -70N and -140D by comparison to the *E. faecalis* sequences.

#### 3.5.1. Structure Modeling

**Figure 7:**
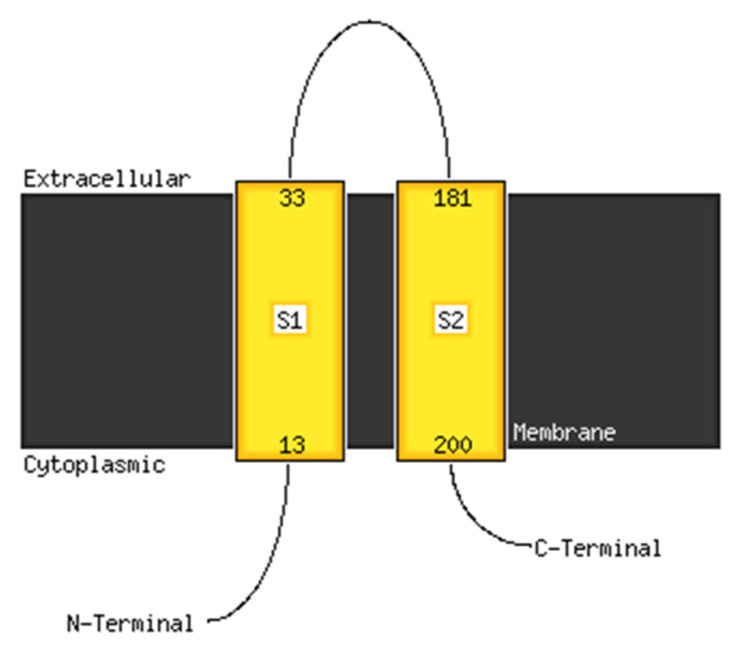
Membrane localization of VicK (WalK) of *E. faecium* NCIMB10415 using the PSIPred server. An extracellular motif is revealed between the amino acid 33 and 181, which may represent the domain binding catecholamines.

The analysis with PSIPRED (analysis performed in October 2020), particularly with the MEMSAT-SVM program, showed that the *E. faecium* NCIMB10415 VicK (WalK) protein consists of two transmembrane regions between amino acids 13 and 33 and between amino acids 181 and 200. The carboxy terminus and the amino terminus were predicted to be in the cytoplasm. Conversely, the sequence between the amino acids 33 and 181 showed to be an extracellular domain, thus representing the region of VicK (WalK) that may interact with the catecholamine ligands (Figure 7). This region will be called VicKex (WalKex) in the manuscript. The analysis with pGenTHREADER and pDomTHREADER—two methods for recognizing and aligning protein sequences—showed significant similarities between the intracellular domain of WalK and several histidine kinases, suggesting its function. Through MODELLER for basic modeling, we aligned the FASTA code of VicKex (WalKex) with the PDB database, and we found a significant alignment with the extracellular domain of sensor histidine kinase YycG from *Staphylococcus aureus* (PDB code: 5IS1) and with the extracellular receptor domain of the sensor kinase WalK from *Staphylococcus aureus* (PDB code: 4YWZ). We then predicted the 3D structure of VicKex (WalKex) by the homology modeling servers RaptorX, GalaxyWeb, and MODELLER through the alignment with YycG and WalK from *S. aureus*. The models thus obtained were then analyzed to determine the optimum predictive model for molecular docking.

#### 3.5.2. Molecular Docking

**Figure 8:**
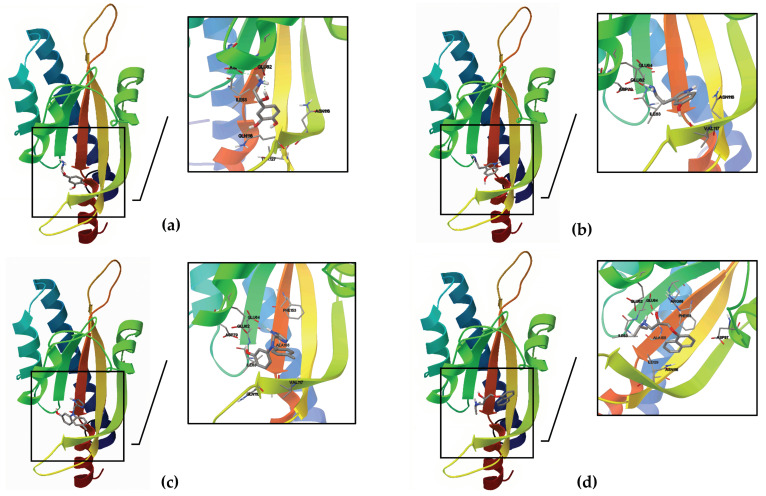
Docking to the putative structure of VicK (WalK) of *E. faecium* NCIMB10415: (**a**) NE; (**b**) 5HT; (**c**) phentolamine; (**d**) propranolol.

A molecular docking investigation was performed using AutoDock 4.2 to see if the *E. faecium* NCIMB10415 VicK (WalK) putative adrenergic sensor would bind NE, 5HT, and the adrenergic blockers phentolamine and propranolol. Each ligand has a total of 50 conformations, each with a specific number of clusters. However, the intermolecular interaction energy function is not always accurate in predicting the actual binding energy because AutoDock considers the enthalpic binding energy in its search for favorable orientations of torsionally flexible ligands; thus, the cluster with the absolute minimum energy does not always represent the best binding site [47]. As a result, the number of alternative conformations for each cluster, the binding energy, and the residues implicated in the binding were used to choose the optimum potential interactions site. Table 2 and Figure 8 illustrate the amino acids involved in the interaction site for the studied ligands, as well as the structure of *E. faecium* NCIMB10415’s VicK (WalK)’s putative binding pocket.

### 3.6. Binding Studies—Micro-Scale Thermophoresis

To complete the in silico study of the putative adrenergic sensor, a Micro-Scale Thermophoresis approach with NE and 5HT was performed to evaluate the binding between *E. faecium* NCIMB10415’s VicK (WalK) and the two hormones. The results obtained by this analysis showed a weak interaction between VicK (WalK) and 5HT, with a dissociation constant (K_D_) of 4.50 ± 0.07 mM. In the same way, NE possessed a very low affinity for VicK (WalK), with a detected binding but a K_D_ that could not be calculated in our condition (MST buffer and concentration of the used labeled VicK (WalK)).

## 4. Discussion

The elucidation of the complex interplay between bacteria and their host has been the object of several reports in recent decades, especially for the so-called microbiota–gut–brain axis [48]. There is increasing interest in understanding the microbial responses to human-derived molecules. However, most studies explore the effects of host bioactive compounds on opportunistic or pathogenic strains, highlighting the risk of increased pathogenicity under stimulation, and data on commensal or probiotics are scarce to date [21]. This aspect becomes even more critical in enterococci, whose controversial nature has been described above.

The present work intends to shed light on the possible modulation that NE and 5HT, two human hormones abundant in the gut, exert on *E. faecium* NCIMB10415 relationships with the host, both at the mucosal and at the immune response level. We therefore analyzed how Caco-2/TC7 cells (as a model of intestinal epithelial cells) and DC (as a model of immune cells) react to the treated and untreated probiotic. These two cell types, permanently involved in the interaction with bacteria, are both responsible for homeostasis at the gut level since the former contribute to gut permeability and the latter to inflammatory responses [49]. Finally, we investigated the presence of a putative surface sensor that could mediate these hormones’ derived effects.

### 4.1. Biofilm, Adhesion and TER

Adhesion on intestinal epithelial cells is considered an essential trait of probiotics, as adherent bacteria may overcome peristaltic movements and permanently colonize the gut epithelium by forming a biofilm structure where they can persist and exert their positive effects on human health [50]. An adhesion-independent biofilm enhancement was previously assessed by crystal violet staining in this strain after treatment with both NE [22] and 5HT [23]. The confocal microscope analysis of the biomasses validated these findings since the biovolumes, and the maximum and average thickness were higher than those under control conditions. Thus, the capability of both molecules to modulate the persistence in the gut ecological niche was confirmed by the adhesion experiment results, although this activity was more pronounced for NE than 5HT. As far as 5HT is concerned, our results, although suggesting a moderate increase in adhesion on enterocytes, were not statistically significant. The only published evidence of 5HT’s effects on bacterial adhesion concerns a 5HT-induced reduction in *C. jejuni* adhesion to colonic epithelial cells [51]. However, because of the taxonomic distance between these two bacterial models, no definitive considerations can be drawn, and further research is required to clarify this poorly explored field. Conversely, the adhesion-boosting effect induced by NE has been described for several commensals or pathogens such as enterohemorrhagic *Escherichia coli*, *P. aeruginosa*, *A. hydrophila*, and *E. faecalis* on different cell lines [21,52,53,54]. In addition, one recent study reported a similar effect induced by NE on two probiotic strains of *E. faecalis* [21].

Adhesion and persistence of beneficial microbes in the GI tract also affect the intestinal barrier function, potentially benefiting the host. In conjunction with the endothelium, the epithelium creates a physiological barrier that controls transport processes to protect tissue physiology and sustain systemic homeostasis [55]. The well-known “leaky gut syndrome” represents the breaking of this balance that can cause severe inflammatory syndromes, ultimately affecting tissue and organs anatomically very distant from the intestine [56]. The epithelium integrity depends on the intercellular junctions (tight junctions, adherent junctions, and desmosomes), while gap junctions are responsible for cell connectivity [57].

Therefore, we evaluated whether untreated, 5HT- and NE-treated bacterial cells could alter the epithelial barrier integrity and permeability (TER), by measuring electrical resistance through a cellular monolayer, as it mirrors the tight junctions’ integrity.

Control *E. faecium* NCIMB10415 enhanced the TER by about 30% compared to the negative control (epithelial cells without bacteria), thus confirming the previous results on the same strain by Lodemann and coworkers [58]. This outcome is not unexpected because probiotic bacteria generally protect the epithelial barrier function by triggering mucus production, tight junctions’ function, and the immune responses also preventing intestinal cells apoptosis [59]. Furthermore, a TER increase has been described after enterocyte treatments with many different probiotics such as *Streptococcus thermophilus*, *Lactobacillus acidophilus*, *Lactobacillus plantarum*, *E. coli* Nissle 1917, and *Bifidobacterium infantis*. Among them, live *S. thermophilus* and *L. acidophilus* increased the TER of HT-29 and Caco-2 cells by inducing the activation of the tight junction’s proteins Occludin and Zonula Occludens (ZO)-1 [60], while *L. plantarum* was shown to trigger the expression of genes related to the tight junction’s formation [61]. In addition, *E. coli* Nissle 1917 stimulated the barrier integrity inducing the expression of ZO-2 proteins [62], while in the secretomes of *B. infantis*, a decreased presence of claudins and increased levels of ZO-1 and occludins via the MAPK pathway were observed [63]. To sum up, the beneficial effects of probiotics on gut integrity can be due either to compounds released in the extracellular compartment or to bacteria surface-adherent proteins. Since we used Caco-2/TC7 cells in close contact with *E. faecium* NCIMB10415, the beneficial effects observed could be due to surface components (increase of 30% of initial TER). However, secreted mediators could also play a role because of the long incubation time (20 h).

Interestingly, pre-treating *Enterococcus* with 100 μM NE and 50 μM 5HT boosted this beneficial impact (increases of 50 and 60% of initial TER, respectively). To our knowledge, there are no reports regarding the TER modification induced by human hormones on probiotics, while some studies have been performed on pathogens. NE boosted the damaging effect on the epithelium of several *Campylobacter* strains such as *C. jejuni* [64,65], *C. coli*, and *C. fetus* subsp. *fetus* on the tight junctions of T84 cells [65]. Epi increased the detrimental effect of *Pseudomonas fluorescens* on Caco-2/TC7 monolayers integrity, while 5HT did not trigger significant effects on it [19]. Thus, we could hypothesize that the hormones abundant in the gut can exert a general stimulating effect on bacteria, whose behavior is ultimately determined by the true nature of the species (pathogen or probiotic). Based on the findings of this research, we can assume that the observed improvement in barrier integrity may be in part related to the increased number of adherent bacteria in the stimulated conditions.

### 4.2. Immune-Targeted Activity

The gut-associated lymphoid tissue (GALT), which constitutes 70% of the overall immune system, is one of the most critical barriers to controlling infection and inflammation [27]. Although the contact between gut bacteria and immune cells is not as direct as that with epithelial cells, specific cell types such as dendritic cells (DC) can protrude in the intestinal lumen and sense bacteria. The contact is mediated by TLRs (Toll-like receptors) on the immune cells and MAMP (microbial-associated molecular patterns) on the bacterial surface. Moreover, small molecules (Short Chain Fatty Acids, amino acids) that can translocate across the gut barrier can be used by bacteria as soluble signals to stimulate immune responses [27].

Several studies demonstrated that probiotic bacteria could influence the host’s immune system and modulate immune responses [28]. Most microorganisms that are considered probiotics are generally selected from the *Lactobacillus* or *Bifidobacterium* genera and have been shown to interact with DC and to induce strain-specific effects. For example, some strains modulate the cytokine production of DC in vitro and induce a regulatory response, while others induce conversely a pro-inflammatory response [66,67]. As far as the genus *Enterococcus* is concerned, the activation of human and murine DC by *E. faecalis* has been shown [29]; however, few data are available about the effects of *E. faecium* on the immune system [30]. The immune-regulating activity of enterococci is an appreciated trait both to counteract infection (immune stimulation) and to control inflammatory bowel syndrome and irritable bowel disease as well as systemic inflammatory pathways (immune modulation) [68,69].

Here, we have evaluated the possible boosting effect that the treatment of *E. faecium* NCIMB10415 with 5HT and NE can have on its natural capability to modulate immune functions using DC as targets. DC are classified into two stages: immature and mature, based on the expression of soluble mediators or unique surface receptors [70]. Some immune parameters such as DC differentiation, maturation, and interleukin (IL) production were considered. First, we observed that DC stimulated with *E. faecium* NCIMB10415, grown in the presence or absence of the two hormones, upregulated differentiation, and maturation markers. Therefore, no significant specific activation due to NE and 5HT stimulation was assessed. The quantification of cytokines secreted in cell culture supernatants was the second objective of our immunological study, and we demonstrated that the strain in this study could modify the release of both pro-inflammatory and anti-inflammatory cytokines by DC.

TNF-α, IL-6 and IL-8 were studied as pro-inflammatory cytokines, and all the *E. faecium* NCIMB10415 cultures (HK/Control, HK/NE, and HK/5HT) were able to induce their release from DC, but to a lesser extent than LPS stimulation. While no effect was observed by 5HT treatment, NE seemed to double up the production of TNF-α (* *p* < 0.05). A significant upregulation of TNF-α production in DC challenged with the simultaneous presence of treated and untreated *E. faecium* NCIMB10415 and LPS was also observed. TNF-α is an inflammatory cytokine that is released during acute inflammation and responsible for a variety of signaling events that lead to necrosis or apoptosis [71].

As far as the anti-inflammatory IL-10 is concerned, all *E. faecium* NCIMB10415’s cultures (HK/Control, HK/NE, HK/5HT) can boost its production at a level comparable to that triggered by LPS, although not significantly. Interestingly, cotreatment with both control and 5HT-stimulated *E. faecium* NCIMB10415 and LPS further enhances the IL-10 production (*p* < 0.01). The latter regulates Th1 and Th2 responses by limiting T cell activation to restrict and terminate inflammatory responses [72]. IL-10 has been related to the immune-modulating activity exerted by probiotics in preventing IBDs [73,74]. These overall findings indicate that *E. faecium* NCIMB10415 can cause specific patterns of cytokine production, particularly IL-10 and TNF-α as shown for many other probiotic LAB strains on different IS cells [67,75]. Furthermore, compared to the Gram-negative-derived LPS, this strain mainly seems to trigger an anti-inflammatory response, even if the pro-inflammatory pathway can also be elicited under NE stimulation.

It is tempting to speculate about the possible molecules produced by *E. faecium* NCIMB10415 and involved in immune cells crosstalk. From one side, we can exclude every secreted compound since the cell-free supernatants did not display any activity on DCs. On the other side, surface structures microbe-associated molecular pattern (MAMPS) are well-known targets for DC interaction [27]. Nevertheless, we must consider that all the treated and untreated cells were HK, and therefore proteins should be excluded because thermal denaturation events can damage protein folding, structure, and performance. Cell-wall components are probably the best candidates for inducing the modulation observed; however, more detailed research is necessary to shed light on these aspects, considering that hormone treatment does not dramatically affect the strain behavior towards DC.

### 4.3. Sensor

Previous studies have identified or hypothesized the presence of bacterial sensors to host molecules, in particular belonging to the family of the TCS, for human hormones in pathogens [21,25,76,77,78]. These systems include an integral trans-membrane kinase (HPK) that acts as a sensor for the stimulus and phosphorylates, plus a response regulator (RR), which mediates the signaling events resulting in gene transcription and targeted expression of specific phenotypic characters in response [79]. In bacterial pathogens, *E. coli* O157:H7 [25] and *Salmonella typhimurium* [76], the two-component systems QseBC and QseEF are involved in response to Epi and NE, and other TCSs have been recently identified in *Citrobacter rodentium* (CpxA—5HT sensor) [78] and in *E. faecalis* VicKR (WalKR)—putative NE and Epi sensor [21].

To investigate whether the probiotic strain *E. faecium* NCIMB10415 possesses a surface receptor responsible for sensing human hormones, we performed both in silico and in vitro studies. A genomic analysis in *E. faecium* NCIMB10415 genome, to search for QseC (the *E. coli* adrenergic receptor) and its homologs VicK (WalK) from *E. faecalis,* also allowed the identification of VicK (WalK) in this bacterium as the nearest protein to QseC with 29% identity and 46% similarity values. This protein is part of the two-component system VicKR (WalKR) also known in the literature as YycGF. Moreover, the whole proteomic analysis performed by our group [23] on *E. faecium* NCIMB10415 treated with NE or 5HT highlighted a higher abundance (fold-change of 1.4) of the DNA-binding response regulator VicR (vicR, HMPREF0351_12362) in 5HT-treated bacteria than in control cultures, supporting the idea of the involvement of the VicKR (YycFG) system in 5HT sensing). However, the VicK component of the TCS was missing since, in the proteome analyses, no enrichment for membrane-bound proteins was performed. On the contrary, treatment with NE did not seem to enhance the production of VicR [22].

Modeling of VicKex (WalKex) structure and molecular docking allowed us to study the interaction of this *E. faecium* NCIMB10415 adrenergic putative sensor with NE and 5HT. Our in silico analysis stands out that NE and 5HT are most likely binding to the receptor, with a free binding energy of −6.35 and −6.12 kcal/mol, respectively. This value found for the interaction between VicK (WalK) and NE is slightly different than the one previously published for *E. faecalis* [21]. This can probably be explained by the indels found in *E. faecium* and substitution of some amino acids in the VicK (WalK) sequence, but also by the models used in the different steps of the molecular docking. We also tested the affinity of two adrenoreceptors antagonists: propranolol and phentolamine. Propranolol showed an intermediate affinity to the receptor (−5.78 kcal/mol). Simultaneously, phentolamine docking showed low binding energy (−3.73 kcal/mol) and many clusters with only one or two conformations, suggesting a non-specific binding that could modify the binding pocket for NE and 5HT and therefore act as a dose-dependent inhibitor. It is also worth mentioning that nearly the same amino acids are involved in the binding of these four molecules to *E. faecium* VicK (WalK).

Finally, the MST analysis detected a weak binding between the purified putative sensor and 5HT with a K_D_ of 4.50 ± 0.07 mM. In contrast with the in silico docking data, NE seemed to also possess a low affinity with a non-measurable K_D_ in our experimental condition. However, some phenotypic changes were detected in the presence of NE, suggesting that this molecule could act through different mechanisms. Two possible hypotheses can support this experimental evidence: (a) NE may enter into the cell via a transport system and elicit its role in the cytoplasm; as a matter of fact, OCT and PMAT-like transports have been reported for other LABs, such as *Lactobacillus* [80]. (b) NE could trigger its cascade effects by means of an intermediate soluble molecule, as for the NE-AI discovered in *E. coli* [81]. It could be argued that the calculated K_D_ for 5HT is too high to justify binding in vivo. However, it has to be considered that Vick (WalK) is a membrane protein, and therefore it is hydrophobic. For this reason, due to the buffer solution, it could be folded in a way not strictly corresponding to its natural conformation. Therefore, it could be interesting, using the MST approach, to test different buffers and detergents to optimize the experimental setting in the near future.

Several studies have proved a connection between the microbial TCS signaling pathway and bacterial responses to different environmental stimuli. For example, it has been shown that QseC, the TCS analog of (VicK) WalK, in *Haemophilus influenzae* [82] and *Aggregatibacter actinomycetemcomitans* [83] controls the biofilm formation. Moreover, WalKR in *S. aureus* proved to be involved in aggregation and biofilm formation [84] as observed in our previous [22,23] and the present work. Therefore, the 5HT stimulation of *E. faecium* NCIMB10415 through the WalK signaling pathway could be responsible for activating pathways linked to the enhancement of aggregating properties and biofilm of the treated bacteria.

We can conclude that our in silico and in vitro experiments neither confirm nor contradict the presence of a sensor on the bacterial surface, and most importantly, do not exclude other different mechanisms of action of the hormones. However, we hope that future investigations can fill the gap adding new consistence to this hypothesis. It is also possible that the VicK/WalK receptor differs from the best-known QseC. Future studies may include the crystallization of the sensor, staining of colocalized molecules and sensors and radioactivity labelling of the hormones to see if they exert their function also by penetrating inside the bacteria.

## 5. Conclusions

In the present study, we demonstrated that the human’s hormones NE and 5HT improved bacterial biofilm formation and increased the percentage of adhesion, albeit not significantly for 5HT, on Caco-2/TC7 epithelial gut cells, thus promoting longer persistence of *E. faecium* NCIMB10415 in the host tissues. Furthermore, the TER experiment intended to evaluate gut cell permeability on differentiated cells demonstrated that the stimulation was beneficial, since the treated bacteria increased the host’s barrier function significantly more than the control cultures of *Enterococcus* NCIMB10415.

Interesting results on the interaction between HK bacteria and DCs were also obtained: even if the hormone treatments had no effect on the strain ability to induce DC maturation and differentiation, the production of the pro-inflammatory TNF-α by DCs challenged with NE-treated HK cells was significantly higher than in control conditions. Moreover, the combined action of LPS and both control and NE-treated HK bacteria boosted the production of TNF-α, whereas the simultaneous presence of LPS and both control and 5HT-treated bacteria increased that of IL-10. These findings, although preliminary (future investigations with living bacteria or different IS cells are required) represent a good starting point since, to the best of our knowledge, this is the first study describing how human bioactive compounds can change the interaction between bacteria and the IS.

In parallel, the results of the investigation on the putative sensor and the previous proteomics data [23] give a hint on the possibility that the hormones could be sensed by these bacteria through VicK (WalK), a membrane receptor that exerts its effect by activating some stress response pathways, enhancing membrane transport and interactive/adhesive capability. However, we cannot exclude that other TCS present on *E. faecium* NCIMB10415 could be involved in the physiological effects observed.

Further studies are required to provide a more comprehensive model for this inter-kingdom signaling and shed light on the possible transition probiotic/pathogen. Finally, the human gut environment, with its complexities and high inter-individual variability, should be carefully considered to fully determine the indirect beneficial or adverse effect of the bacterial stimulation on the host physiology.

## Figures and Tables

**Table 1:** Effect of the treatment of *E. faecium* NCIMB10415 with 100 µM NE and 50 µM 5HT for 48 h on biofilm formation (biovolume, average thickness, and maximum thickness) on glass surface, measured by CLSM. Both treatments increase the biofilm volume and thickness. The results are expressed ± SD *** *p* ≤ 0.001, ** *p* < 0.01, * *p* < 0.05.

Treatment	Biovolumes (µm^3^/µm^2^)	Average Thickness (µm)	Maximum Thickness (µm)
Control	0.63 ± 0.27	1.4 ± 0.8	13.2 ± 2.3
100 µM NE	0.98 ± 0.28 **	2.3 ± 1.0 *	18.6 ± 4.1 ***
50 µM 5HT	1.24 ± 0.48 **	3.0 ± 1.2 ***	18.4 ± 4.1 ***

**Table 2:** The binding energy, number of conformations in the selected cluster, and the amino acids involved in the interaction site of VicK (WalK) for the four ligands tested.

Molecule	Binding Energy (kcal/mol)	Number of Conformations	Amino Acids Involved
NE	−6.35	7	ILE63, ASP78, GLU82, ASN116, GLN118, THR127
5HT	−6.12	5	ILE63, GLU64, ASP79, GLU82, ASN116, VAL117
Phentolamine	−3.73	2	ILE63, GLU64, ASP79, GLU82, VAL117, GLN118, PHE153, ALA155
Propranolol	−5.78	4	ILE63, GLU64, ARG66, GLU82, ASP97, ASN116, ILE129, PHE153, ALA155

## Data Availability

Not Applicable.

## Supplementary Materials

The following are available online at https://www.mdpi.com/article/10.3390/microorganisms10030487/s1, Figure S1: TER measurements over time.
