# Supplementary material for: Norepinephrine and Serotonin Can Modulate the Behavior of the Probiotic Enterococcus faecium NCIMB10415 towards the Host: Is a Putative Surface Sensor Involved?
Source: Microorganisms. 2022 Feb 22;10(3):487. doi: 10.3390/microorganisms10030487 (PMC8954575; doi:10.3390/microorganisms10030487)
Supplement: Supplementary file 1 [file microorganisms-10-00487-s001.zip › microorganisms-1560297-supplementary.pdf]

Supplementary Figure: TER measurements over time (T_4 T_{16} T_{20})

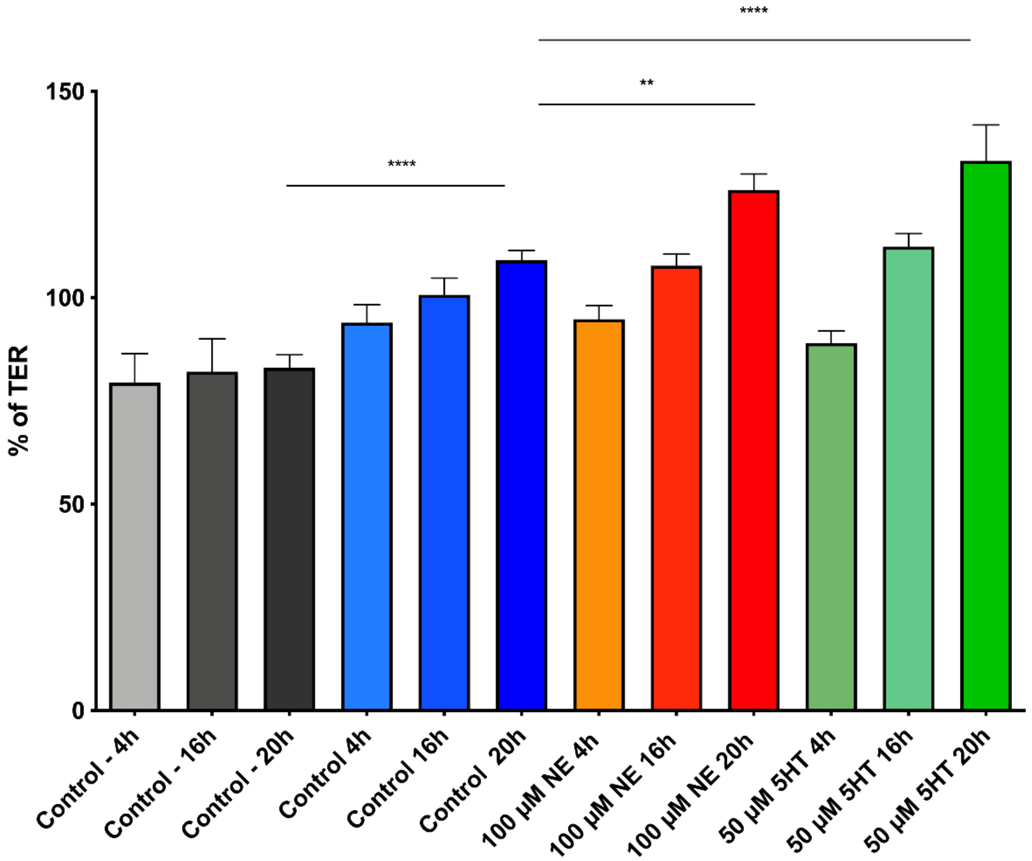

Supplementary Figure S1. Modulation of Caco-2/TC7 TER after 4, 16 and 20 h exposure to *E. faecium* NCIMB10415 in control conditions (shades of blue) or treated with 100 µM NE (shades of red) or 50 µM 5HT (shades of green). The figure also displays how the TER recordings changed over time in negative control (not treated with bacteria, Control – , shades of grey). Results are expressed as % of initial TER ± SEM. *E. faecium* NCIMB10415 (control and treatments) starts to slightly increase the TER already after 4 hours, even if not significantly; the highest difference with Control - is observed after 20 hours. Interestingly, both hormones' stimulations of the bacteria increased the TER compared to the control condition (blue). **** $p \leq 0.0001$ *** $p \leq 0.001$, ** $p < 0.01$.
